# A Superhydrophilic Au-Coated Monolayer Polystyrene Sphere Substrate for Uniform Surface-Enhanced Raman Spectroscopy

**DOI:** 10.1021/acsomega.5c08273

**Published:** 2026-04-16

**Authors:** Kittidhaj Dhanasiwawong, Kruawan Wongpanya, Tossaporn Lertvanithphol, Sakdinan Jantarachote, Kanin Aungskunsiri, Mati Horprathum

**Affiliations:** 54782 National Electronics and Computer Technology Center, 112 Thailand Science Park, Phahonyothin Road, Khlong Nueng, Khlong Luang, Pathum Thani 12120, Thailand

## Abstract

Uniform molecular dispersion and strong signal enhancement are critical for reproducible surface-enhanced Raman spectroscopy (SERS). However, conventional SERS substrates often suffer from poor wettability, leading to nonuniform analyte deposition and signal fluctuation. Herein, we present a superhydrophilic SERS substrate composed of gold-coated monolayer polystyrene spheres (Au-MPS), fabricated via doctor-blade-assisted colloidal lithography, followed by gold sputtering. The resulting Au-MPS exhibits both excellent wettability and tunable plasmonic properties. Optical, morphological, and spectroscopic characterizations reveal distinct plasmonic coupling behavior, with a maximum enhancement factor of ∼10^5^-fold and a limit of detection down to the micromolar range using Rhodamine 6G as a probe molecule. The scalable fabrication and consistent performance highlight the potential of this substrate for cost-effective and practical sensing applications. Furthermore, detecting trace levels of the toxic paraquat herbicide is demonstrated, indicating the potential for real-world applications.

## Graphical abstract

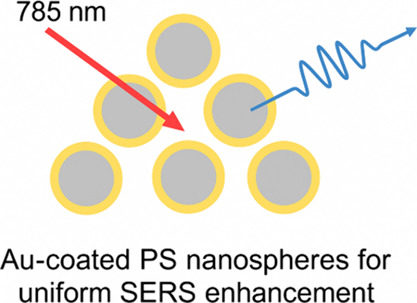

## 1 Introduction

Surface-enhanced Raman spectroscopy (SERS) is a powerful analytical technique for trace-level detection of biological and chemical species, offering high sensitivity, label-free operation, and real-time analysis capabilities. Its performance largely depends on two critical factors: (i) the plasmonic properties of the nanostructured substrate^1^ and (ii) the uniform dispersion of target molecules on the sensing surface.^2^

To enhance SERS sensitivity, various strategies have been employed to optimize the nanostructure design. Huang et al.^1^ demonstrated that reducing the tip spacing of gold nanopillars increases plasmonic hotspots and significantly amplifies Raman signals. Pérez-Jiménez et al.^2^ provided a comprehensive review on material selection and nanostructure engineering for effective SERS platforms. Among the scalable fabrication approaches, colloidal lithography has gained traction for producing ordered arrays of metallic nanostructures with tunable optical properties.^3,4^

Beyond nanostructural design, substrate wettability plays a pivotal role in ensuring a uniform analyte coverage. Traditional SERS substrates often exhibit poor hydrophilicity, leading to analyte aggregation and spatial signal variation due to the coffee-ring effect. To address this challenge, Wang et al.^5^ reported a superhydrophilic SERS substrate that minimizes this phenomenon, enabling consistent molecular adsorption. However, such approaches typically rely on multistep fabrication processes involving wet-chemical synthesis, repeated purification steps, and prolonged processing times, which can increase production costs and limit scalability, even when using conventional plasmonic materials.

Recent advancements have thus shifted toward integrating plasmonic tunability with superhydrophilic and flexible substrate designs to improve the sensing uniformity and expand application versatility. For example, a wearable SERS sensor based on a silver nanowire-coated PTFE substrate has demonstrated uniform signal distribution for on-skin, real-time chemical detection.^6^ Likewise, Das et al. developed a flexible SERS platform using Ag/Au-coated inverted nanopyramid arrays capable of stable hemoglobin detection on skin-like substrates, achieving enhancement factors in the range of 10^5^–10^6^ even under mechanical deformation.^7^

Among various SERS substrate architectures, gold-coated polystyrene spheres have shown great promise by simultaneously offering tunable plasmonic properties and scalable fabrication. Adjusting the sphere diameter and deposition parameters allows precise control over the localized surface plasmon resonance (LSPR), contributing to consistent and enhanced Raman signals. Wang et al.^8^ demonstrated that gold film deposition over polystyrene spheres significantly improved SERS performance by increasing hotspot density via large-area, cost-effective fabrication. Further, Liu et al.^9^ introduced a porous Au nanoparticle array with dense built-in hotspots that consistently provided strong Raman enhancements across the substrate.

In parallel, the use of Fano resonances has emerged as an effective mechanism for amplifying the electromagnetic field intensities in SERS systems. Zhu et al.^10^ reported a plasmonic nanoparticle-in-cavity nanoantenna array exhibiting dual Fano resonances that facilitated cascaded field enhancement and superior SERS sensitivity. Similarly, Wang et al.^11^ developed a metal–insulator–metal (MIM) system comprising coupled heterocavities with independently tunable Fano modes, enabling refined spectral selectivity and resonance control. These innovations highlight the potential of advanced photonic designs for next-generation tunable SERS platforms.

Building upon these concepts, this study introduces a novel SERS substrate based on gold-coated monolayer polystyrene spheres (Au-MPS). The substrate is fabricated using a doctor-blade-assisted colloidal lithography process followed by gold sputtering, resulting in a structure that simultaneously exhibits superhydrophilicity and tunable plasmonic characteristics. This work aims to demonstrate the substrate’s high Raman enhancement performance and its applicability for sensitive chemical and biosensing platforms.

## 2 Methods

### 2.1 Preparation of Au-MPS Samples

The process for preparing the Au-MPS sample is illustrated in Figure 1 and is described as follows.

**Figure 1:**
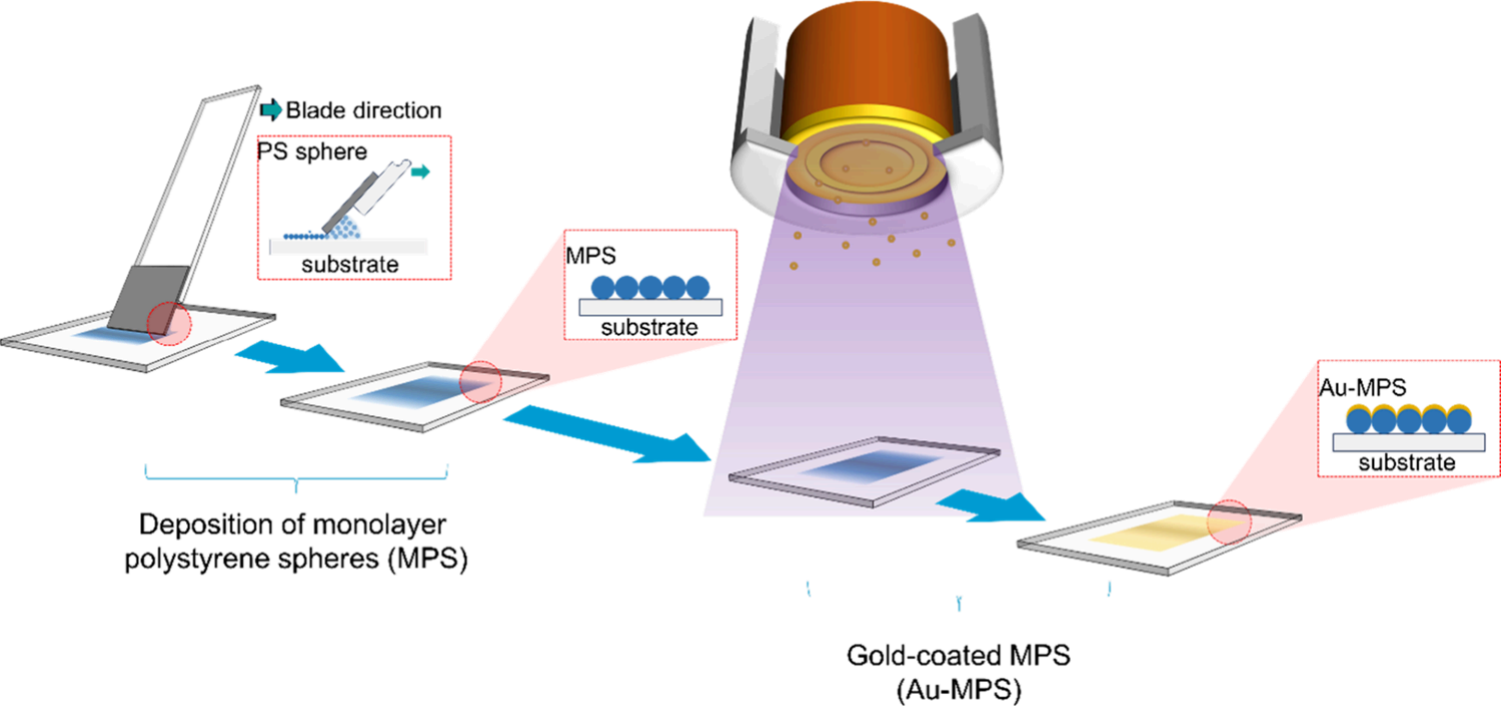
Au-MPS fabrication process (all images in this figure were created by the authors and have not been published previously).

First, a glass substrate was treated with oxygen (O_2_) plasma to improve its hydrophilicity. A monolayer of polystyrene spheres was then prepared on the substrate by using the doctor-blade coating technique. A 20 μL aliquot of the sphere aqueous suspension (1% (w/v) PS, Duke Scientific Corporation, USA) was dropped onto the substrate surface. The average diameters of the spheres varied at 500, 700, 800, and 1000 nm. During the doctor-blade coating process, the temperature and relative humidity were maintained at 25 °C and 55% RH, respectively. The blade’s translation speeds were optimized at 3.00, 3.53, 3.75, and 4.28 μm/s for spheres with diameters of 500, 700, 800, and 1000 nm, respectively. The blade angle, blade–substrate distance, and vibration frequency were fixed at 45°, 0.1 mm, and 240 Hz, respectively, as reported in our previous work.^12^ The reproducibility of the fabricated film substrates was evaluated by preparing five independent films under identical coating and drying conditions. The optical uniformity was analyzed using ImageJ within a 7 × 7 mm^2^ central region of each film. The detailed procedures and the corresponding data are provided in the Supporting Information (Figure S1).

Finally, a thin Au film was deposited onto the monolayer samples by using sputtering. During the sputtering process, the Ar flow rate, chamber pressure, and bias power were maintained at 20 sccm, 5 mTorr, and 100 W, respectively. The adatom flux was oriented perpendicular to the sample surface, and the thickness of the Au film was set to 75 nm.

### 2.2 Au-MPS Characterizations

The morphology of the monolayer polystyrene spheres (MPS) was characterized using an optical microscope (Leica DMC5400) at a magnification of 5× to calculate the monolayer coverage within a 7 × 7 mm^2^ area on the glass substrate using image processing. After gold coating, surface-view field-emission scanning electron microscopy (FE-SEM; SU8030, Hitachi High-Tech) was performed to examine the structural defects, hexagonal close-packed (HCP) structure, and size and spacing of the spheres in the Au-MPS samples. The thickness of the Au film was determined using transmission electron microscopy (TEM; JEM-2010, JEOL). The wettability of the Au-MPS surface was evaluated by using a contact angle goniometer (model 250, ramé-hart). A 6 μL water droplet was placed on the sample surface, and the water contact angle (WCA) was measured at room temperature. The optical absorbance of all samples was obtained using a UV–vis–NIR spectrophotometer (Cary 7000, Agilent) over the spectral range of 400–1600 nm with a 1 nm resolution. All measured absorbance spectra were subsequently normalized to emphasize the spectral shape and plasmonic resonance modes, enabling a direct comparison with the simulated results.

### 2.3 Simulation Methodologies

Numerical simulations were performed using the finite-element method (FEM) in COMSOL Multiphysics^13^ to investigate the plasmonic coupling and absorption behavior of the Au-MPS. A 3D unit cell model of an HCP monolayer of Au-coated spheres was constructed. Floquet periodic boundary conditions were applied along the lateral directions to represent the infinite array periodicity, while ports were defined at the top and bottom boundaries to represent normally incident plane waves propagating along the *z*-axis. Perfectly matched layers (PMLs) were included above and below the computational domains to eliminate spurious reflections.

The incident light was modeled as a periodic plane wave covering wavelengths from 400 to 1600 nm (corresponding to 187.4–749.5 THz) under normal incidence. The simulated geometry, shown in Figure S1 (Supporting Information), consisted of Au-coated PS spheres arranged in a hexagonally packed monolayer on a 50 nm-thick SiO_2_ adhesion layer deposited on a quartz substrate. The PS bead diameters used in this work were 500, 700, 800, and 1000 nm.

The optical constants of Au were obtained from Johnson and Christy.^14^ The refractive index of polystyrene was modeled using the Cauchy-type dispersion relation reported by Zhang et al.^15^ The refractive indices of SiO_2_ and quartz (substrate) were set to 1.46 ^16^ and 1.45,^17^ respectively. A fine tetrahedral mesh was employed, with element sizes refined to ≤5 nm at the Au-air and Au-PS interfaces to accurately capture the localized field enhancement within nanogaps. The frequency-domain solver was used to compute steady-state electromagnetic fields.

The reflectance (*R*) and transmittance (*T*) were extracted from the complex *S*-parameters at the top and bottom ports, and the absorbance (*A*) was obtained from

A=1−R−T 1

The spatial distribution of the electric-field intensity (|*E*|^2^) was analyzed to identify localized surface plasmon (LSP) and Fano-type hybrid resonance modes within the Au–PS sphere array.

### 2.4 SERS Performance of Au-MPS Samples

The Au-MPS samples were used as SERS substrates. Their performance was evaluated using a standard dye solution of Rhodamine 6G (R6G). To prepare the test samples, R6G was dissolved in deionized (DI) water at concentrations ranging from 10^–3^ to 10^–7^ M. A 2 μL droplet of each concentration was placed on the Au-MPS samples prepared using MPS with diameters of 1000, 800, 700, and 500 nm and allowed to dry at room temperature. The Raman spectra were recorded using a confocal Raman spectrometer (Renishaw inVia) with a 50× objective lens, a 785 nm excitation wavelength, a laser power of 1.8 mW, and an exposure time of 10 s. The 785 nm excitation wavelength was chosen to minimize background noise and ensure the most reliable signal-to-noise ratio for quantitative SERS analysis (as shown in Figure S2 (Supporting Information)). The Raman signal intensities were used to calculate the enhancement factor (EF) and limit of detection (LOD) for each sample. In addition, individual measurements were performed at 10 points on the sample’s surface; the signals were then averaged to determine the relative standard deviation (RSD). The stability and reusability of the fabricated film substrate were examined by using R6G as a standard analyte. The same film was subjected to 10 successive measurements under identical experimental conditions. After each measurement, the film surface was rinsed with deionized water, dried, and reused. The signal intensity obtained from each run was recorded to evaluate the stability of the substrate. The real-world application of the fabricated film was verified by using the herbicide paraquat as a target analyte.

## 3 Results and Discussion

### 3.1 Physical Characterizations of Au-MPS

The fabricated MPS samples with various diameters are initially characterized using an optical microscope at 5× magnification, as shown in Figure 2. The acquired images are converted to monochrome to facilitate the identification of monolayer and multilayer regions within the 7 × 7 mm^2^ observation area (outlined by the dashed yellow square). Based on image analysis, the monolayer coverage is determined to be 90.54%, 86.25%, 83.82%, and 90.85% for samples prepared with PS sphere diameters of 1000, 800, 700, and 500 nm, respectively.

**Figure 2:**
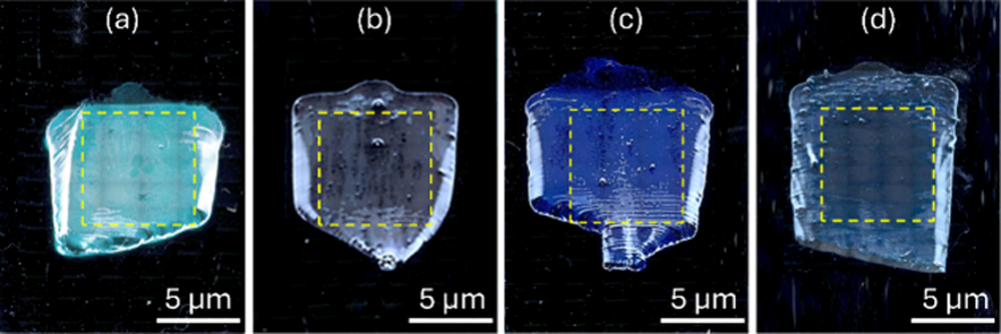
Images of MPS deposited on glass substrates with diameters of (a) 1000 nm, (b) 800 nm, (c) 700 nm, and (d) 500 nm. All images are captured at 5× magnification. The dashed yellow squares indicate the 7 × 7 mm^2^ regions used for monolayer coverage analysis (all images in this figure were created by the authors and have not been published previously).

The high-coverage monolayer samples are subsequently coated with a gold thin film to form the Au-MPS structures and examined in surface view by using FE-SEM, as shown in Figure 3. The spheres assemble into a compact monolayer with an HCP arrangement, as illustrated in the inset images. Some structural defects, highlighted in red, are also observed. These defects classified as line and point defects arise from the polycrystalline nature of the colloidal assembly, which inherently favors defect formation.^18^ Line defects typically result from thermal mismatch between the substrate and the colloidal film, where volume shrinkage of the water layer during self-assembly causes discontinuities.^19,20^ In contrast, point defects can form due to cooperative displacements among colloids, particularly when individual particles exhibit relatively low bonding energy compared to their neighbors, leading to vacancies.^21^ From three independent regions of each sample, the average HCP-to-defect ratios are determined to be 7.6:2.4, 7.9:2.1, 7.7:2.3, and 8.7:1.3 for sphere diameters of 1000, 800, 700, and 500 nm, respectively. Detailed measurements, including the sphere size, Au-MPS diameter, and interparticle spacing, are summarized in Table 1. The reproducibility of the film substrates was 88.92% (as shown in Figure S3 (Supporting Information)), demonstrating the reliability of the coating process under practical fabrication conditions.

**Figure 3:**
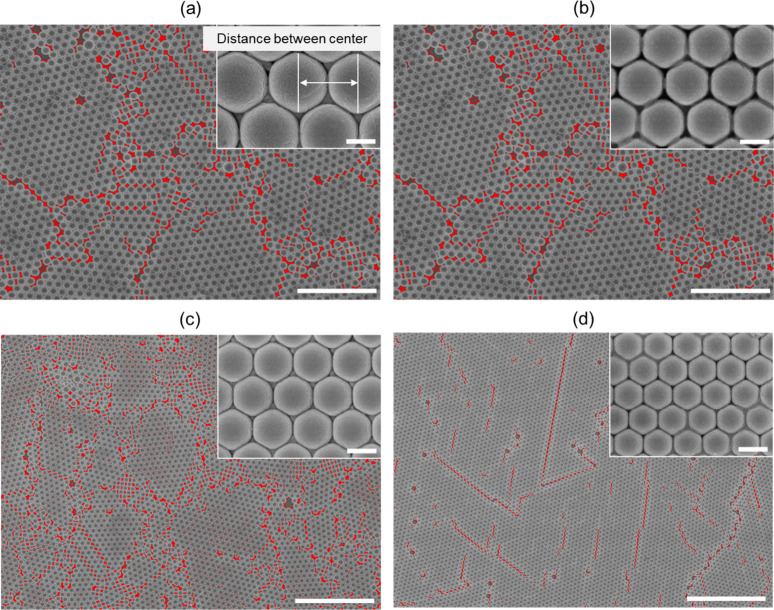
Surface-view FE-SEM images of Au-MPS samples prepared on glass substrates using polystyrene spheres with diameters of (a) 1000 nm, (b) 800 nm, (c) 700 nm, and (d) 500 nm. All main images are shown with 10 μm scale bars. The insets display high-magnification FE-SEM images highlighting the HCP structure and defect regions, with scale bars of 500 nm.

**Table 1:** Summary of Structural Characteristics of Au-MPS Samples Prepared with Different Polystyrene Sphere Diameters, Including the Measured Au-MPS Diameter, Interparticle Center-to-Center Distance, Monolayer Coverage, and HCP-to-Defect Ratio

polystyrene sphere size	Au-MPS diameter	distance between centers	monolayer coverage	HCP-to-defect ratios
1000 nm	1037 ± 38 nm	1050 ± 7 nm	90.54%	7.6:2.4
800 nm	816 ± 15 nm	821 ± 8 nm	86.25%	7.9:2.1
700 nm	725 ± 29 nm	748 ± 9 nm	83.82%	7.7:2.3
500 nm	519 ± 19 nm	529 ± 4 nm	90.85%	8.7:1.3

The cross-sectional morphology of the Au-MPS sample fabricated with 1000 nm polystyrene spheres is examined using FE-SEM, as shown in Figure 4a. The results reveal that the sputtered gold film forms a dome-like structure covering the upper hemisphere of each sphere. Additionally, a partial gold coating is observed on the glass substrate beneath the spheres. This deposition pattern is attributed to the shadowing effect inherent in the sputtering process. Specifically, when the adatom flux is oriented perpendicular to the sample surface, the spheres create shadowed regions where direct coating is obstructed. However, voids within the HCP structure allow some adatoms to penetrate through the interparticle gaps and deposit onto the underlying substrate.

**Figure 4:**
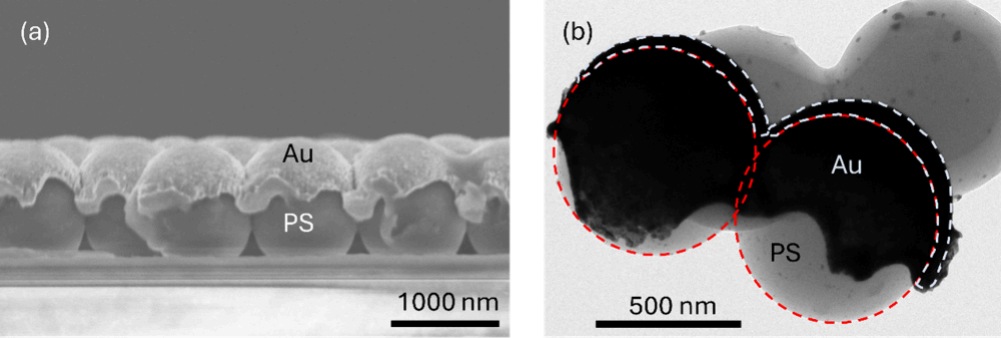
(a) Cross-sectional FE-SEM image of a Au-MPS sample prepared using 1000 nm polystyrene spheres and (b) TEM image of a Au-MPS sample prepared using 700 nm polystyrene spheres for gold thickness estimation.

The thickness of the gold layer on the spheres was estimated from the TEM image of the Au-MPS sample fabricated with 700 nm polystyrene spheres, as shown in Figure 4b, and was determined to be 75 ± 2 nm. This thickness was selected because it exhibits the most well-defined LSPR features, ensuring optimal plasmonic coupling with the excitation wavelength (as shown in Figure S4).

The wettability of both Au film and Au-MPS samples is evaluated by using water contact angle (WCA) measurements. The Au film exhibits a WCA of 90.1°, indicating its hydrophobic behavior. In contrast, the Au-MPS surface displays superhydrophilic properties with rapid droplet spreading, an initial WCA of 7.5°, and complete drying within 1 min of application.^22^ Representative contact angle micrographs are provided in Figure S5.

This unexpected wettability contrasts with the anticipated superhydrophobicity of the surface given that both polystyrene spheres and conventional gold nanostructures are typically hydrophobic. The superhydrophilic behavior observed in the Au-MPS sample is likely attributed to its structural morphology. FE-SEM analysis reveals the presence of nanogaps within the hexagonally close-packed structure and along defect lines, where the gold coating is incomplete. These gaps may serve as capillary channels, enabling water molecules to penetrate through the sphere layer and interact directly with the underlying glass substrate, which is intrinsically hydrophilic. Once contact is established, the cohesive force among water molecules exceeds the adhesive interaction between water and the Au-MPS surface, resulting in rapid spreading. The substrate thereby pulls the droplet through the nanogaps, facilitating isotropic wetting across the surface.

It should be noted that the contact angle of the planar Au film was measured immediately after the sputtering process under ambient conditions identical with those used for the Au-MPS sample. The hydrophobicity of the flat Au film is likely caused by the rapid adsorption of airborne organic species on the gold surface, as commonly reported in the literature. In contrast, the superhydrophilic behavior of the Au-MPS sample originates from its structural morphology, in which nanoscale gaps and defect lines act as capillary channels that allow water to penetrate through the Au-coated sphere layer and interact with the underlying hydrophilic glass substrate.

The normalized optical absorbance spectra of the Au-MPS samples, as shown in Figure 5, exhibit clear Fano-like resonance features, particularly for structures prepared with 700–1000 nm polystyrene spheres. The same spectral features are also observed in the non-normalized absorbance spectra provided in Figure S6, confirming that the observed resonance behavior is not an artifact of normalization. These asymmetric peaks and dips arise from the interference between bright dipolar (super-radiant) and dark quadrupolar (subradiant) collective modes supported by the HCP array. Spectral positions 1 and 3 correspond to dark modes, where the net plasmonic polarization of the peripheral nanoparticles oscillates out of phase with the central one, whereas position 2 represents an in-phase bright collective mode. The interference between these modes produces the characteristic Fano-type resonance dip within the broader absorption band.^23–25^

**Figure 5:**
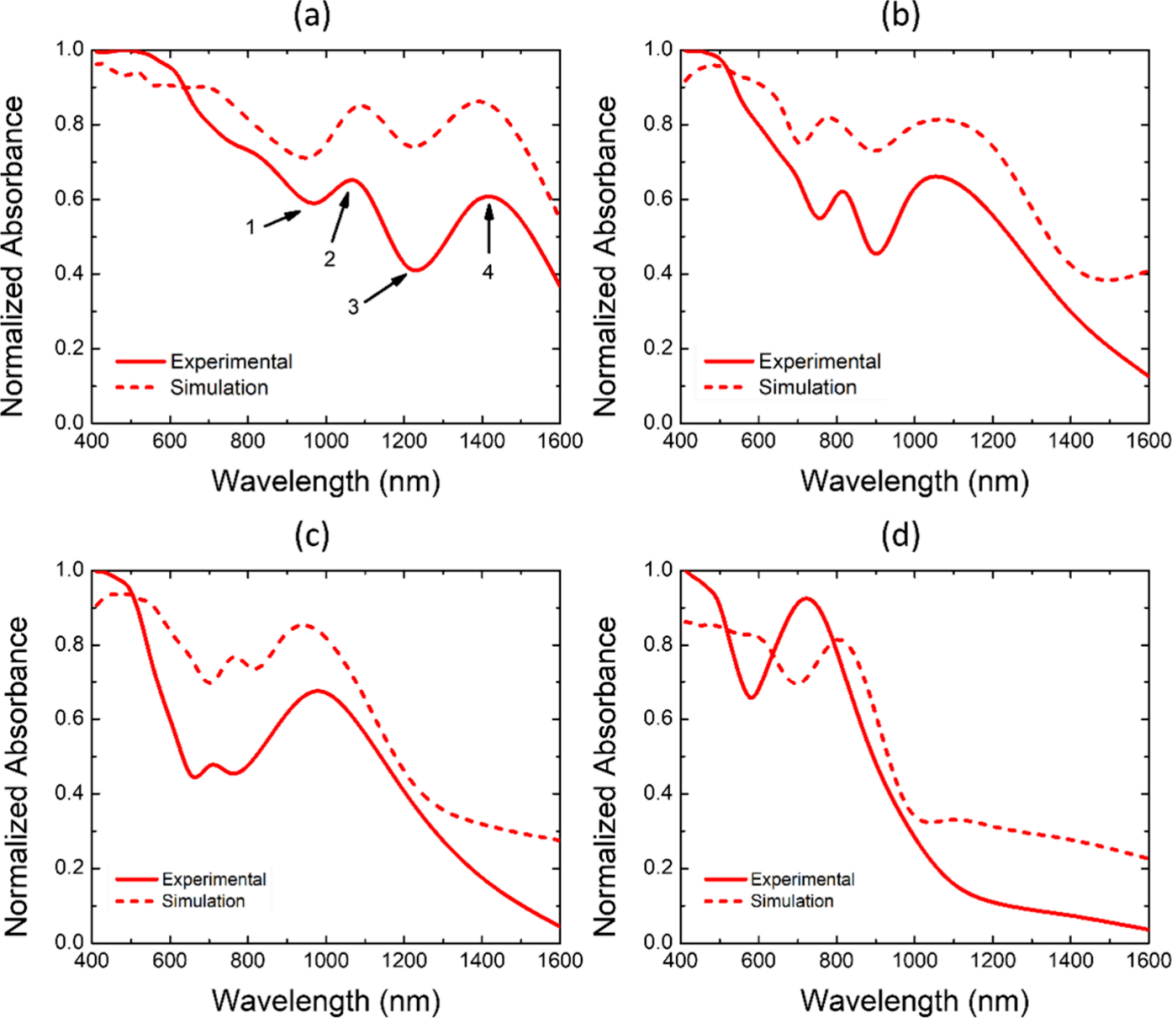
Experimental (solid lines) and simulated (dashed lines) optical absorbance spectra of Au-MPS samples fabricated using PS with diameters of (a) 1000 nm, (b) 800 nm, (c) 700 nm, and (d) 500 nm.

The simulated spectra (dashed lines) reproduce the main experimental trends, confirming the presence of multiple plasmonic coupling modes within the Au–PS array. The experimental peaks appear broader and slightly redshifted, attributable to surface roughness, nanogap variation, and morphological deviations from the ideal model. An additional feature at position 4 arises from the plasmonic response of the underlying Au film, which blueshifts with a decreasing sphere diameter due to changes in effective film thickness and coupling between localized and propagating modes.^26,27^

In contrast, the Au-MPS sample prepared with 500 nm spheres shows no distinct bright super-radiant mode. The broad Au film response likely overlaps with a weak Fano resonance, resulting in a merged and less-defined absorption profile across the visible–near-infrared region.

Figure 6 presents the simulated electric-field intensity (|*E*|^2^) distributions for Au-MPSs with different sphere diameters under a 785 nm excitation. The 700 nm PS-based structure exhibits the strongest |*E*|^2^ localization at the interparticle nanogaps, consistent with the highest Raman enhancement observed experimentally at this wavelength. This confirms that the resonance condition of the 700 nm structure provides optimal coupling for localized surface plasmon excitation at 785 nm, thereby enhancing the SERS performance.

**Figure 6:**
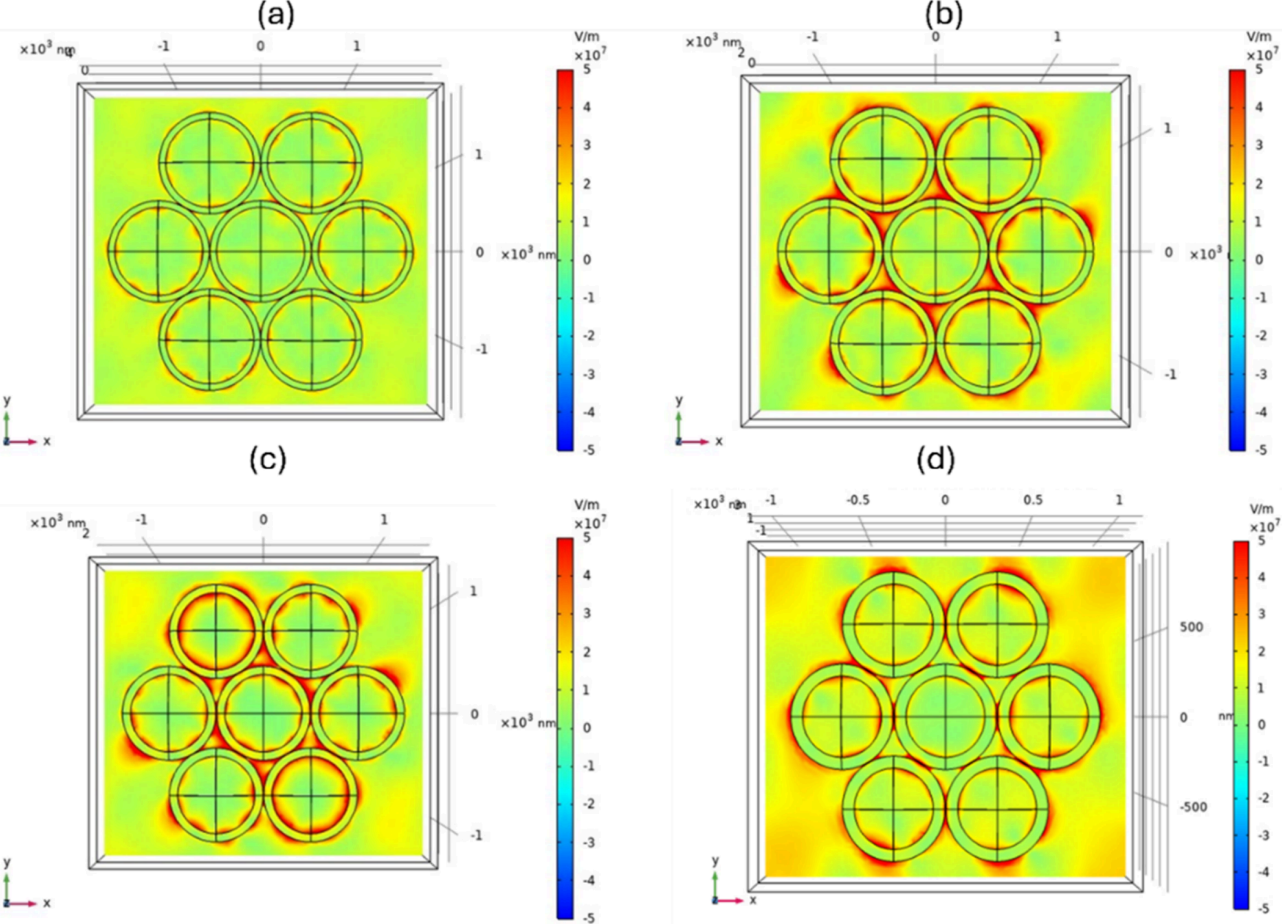
Simulated electric-field intensity (|*E*|^2^) distributions of Au-MPS structures with PS diameters of (a) 1000 nm, (b) 800 nm, (c) 700 nm, and (d) 500 nm under 785 nm excitation.

### 3.2 SERS Performance of Au-MPS

To evaluate the SERS performance, dried R6G solutions at concentrations ranging from 10^–3^ to 10^–7^ M are deposited onto both the Au film and Au-MPS substrates and subsequently measured for Raman spectra at 10 points on the SERS surface and then averaged as shown in Figure 7a–e. The recorded spectra exhibit several characteristic peaks of R6G, including C–H stretching (1186 cm^–1^), C–O–C in-plane bending within the aromatic ring (1310 cm^–1^), and C–C stretching vibrations (1362, 1510, and 1650 cm^–1^).^28^

**Figure 7:**
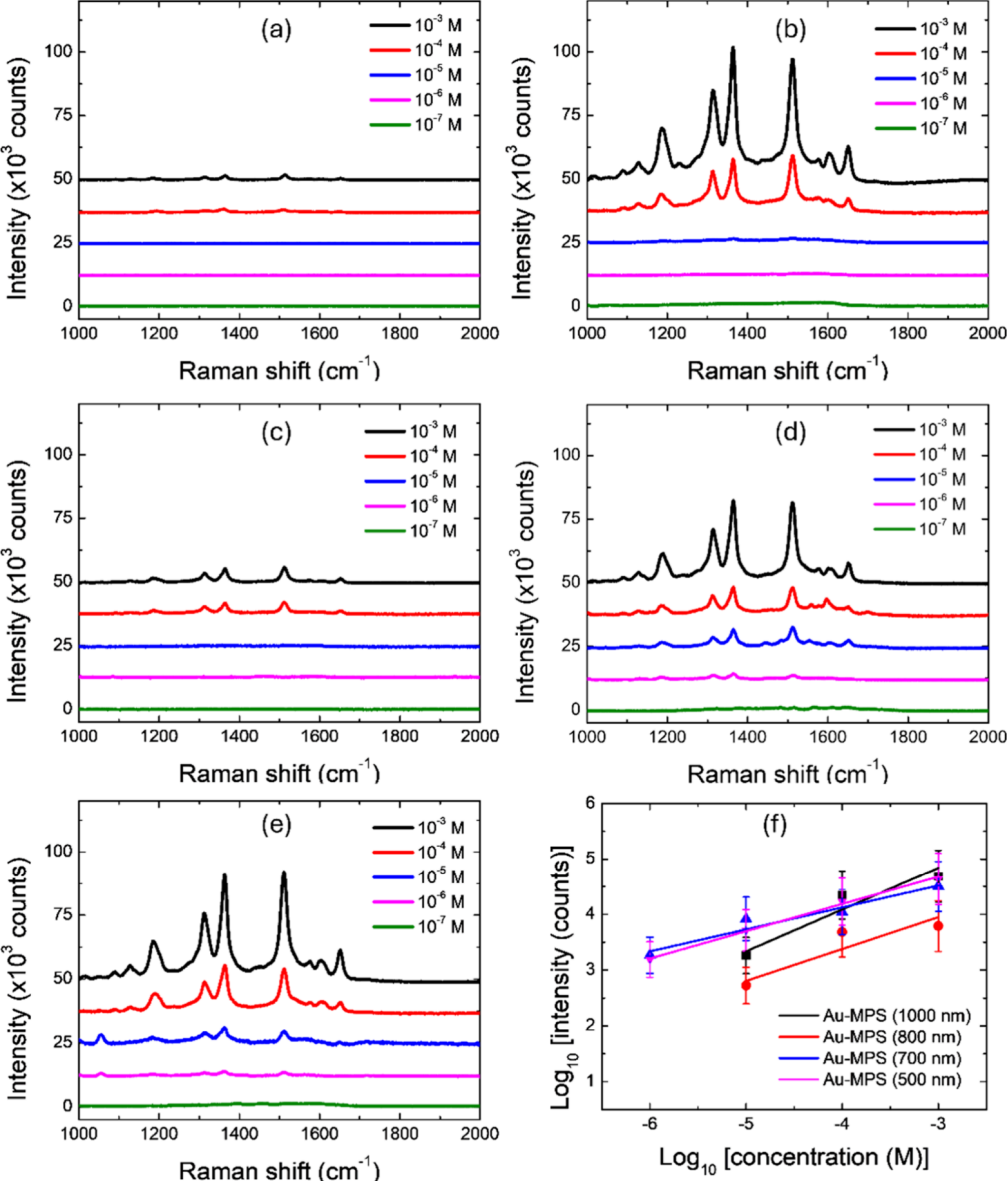
Raman spectra of R6G (10^–3^–10^–7^ M) measured on (a) flat Au and Au-MPS substrates with PS diameters of (b) 1000 nm, (c) 800 nm, (d) 700 nm, and (e) 500 nm. (f) Log–log plots of Raman intensity at 1510 cm^–1^ versus concentration for LOD determination.

For the Au film, R6G peaks are clearly observed at 10^–3^ and 10^–4^ M, with weak signals detectable at 10^–5^ M. In contrast, the Au-MPS samples yield distinct R6G peaks at concentrations below 10^–4^ M, indicating superior Raman enhancement and a lower LOD compared to the planar Au film.

To quantitatively assess the SERS performance, the average Raman intensities at 1310, 1362, and 1510 cm^–1^ are calculated and plotted on a logarithmic scale across different concentrations, as shown in Figure 7f. The LOD is estimated from the linear fitting using the equation:

LOD=3.3σ/slope 2

where σ denotes the standard deviation of the Raman intensity at the lowest detectable concentration.^29^ The calculated LOD values for Au-MPS samples prepared with 1000, 800, 700, and 500 nm spheres are 2.69 × 10^–6^, 2.45 × 10^–5^, 2.77 × 10^–7^, and 4.04 × 10^–7^ M, respectively. Among these, the sample with 700 nm spheres achieves the lowest LOD and strongest Raman enhancement, with R6G peaks clearly visible even at 10^–7^ M. Experimentally, although the 800 nm Au-MPS exhibits pronounced plasmonic features in the optical absorbance spectrum, its SERS performance is weaker than that of the 700 nm array. This behavior is likely attributed to partial detuning between the dominant plasmonic mode of the 800 nm structure and the 785 nm excitation wavelength. In addition, the 800 nm array may be affected by variations in sphere packing that are not considered in the simulations, which can reduce the effective hotspot coupling and lead to weaker experimental SERS signals.

The enhancement factor (EF) for the Au-MPS sample with 700 nm spheres identified as the optimal substrate based on its lowest LOD is calculated using the following standard formula:^30^

$$\mathrm{EF}=(I_{\mathrm{Au}\mathrm{‐MPS}700}/I_{\mathrm{Au}\;\mathrm{film}})\times (N_{\mathrm{Au}\mathrm{‐MPS}700}/N_{\mathrm{Au}\;\mathrm{film}})$$ 3

where *I* represent the Raman intensity and *N* denotes the estimated number of R6G molecules within the laser spot for each substrate. The calculated EF values at various concentrations are summarized in Table 2. Notably, the highest EF is observed at 10^–5^ M, while values at lower concentrations cannot be determined due to undetectable signals from the Au film reference.

**Table 2:** Enhancement Factors of the Au-MPS Substrate Fabricated with 700 nm Polystyrene Spheres, Calculated at Selected Raman Peak Positions for Various R6G Concentrations

	EF at a peak position (×10^3^-fold)		
R6G concentration (M)	1310 cm^–1^	1362 cm^–1^	1510 cm^–1^
1 × 10^–3^	39.38	43.79	42.98
1 × 10^–4^	23.51	22.76	24.71
1 × 10^–5^	81.18	121.17	141.70

To evaluate the signal uniformity of the R6G molecules on the Au-MPS substrate, point-by-point Raman measurements were systematically performed. The Raman spectra related to 10 different areas of the 10^–4^ M R6G on Au-MPS with 700 nm polystyrene spheres are represented in Figure 8a,b. The relative standard deviation (RSD) of the peak intensity located at 1510 cm^–1^ was calculated as 6.83%, as shown in Figure 8c. The results indicate that the Au-MPS substrate has good uniformity across the sample for SERS applications. Additionally, the stability of the fabricated film was evaluated by repeatedly measuring the SERS signal of 10^–5^ M R6G 10 times on the same substrate and re-evaluating it after 12 months of storage, yielding an RSD of 10.19% (Figure S7, Supporting Information).

**Figure 8:**
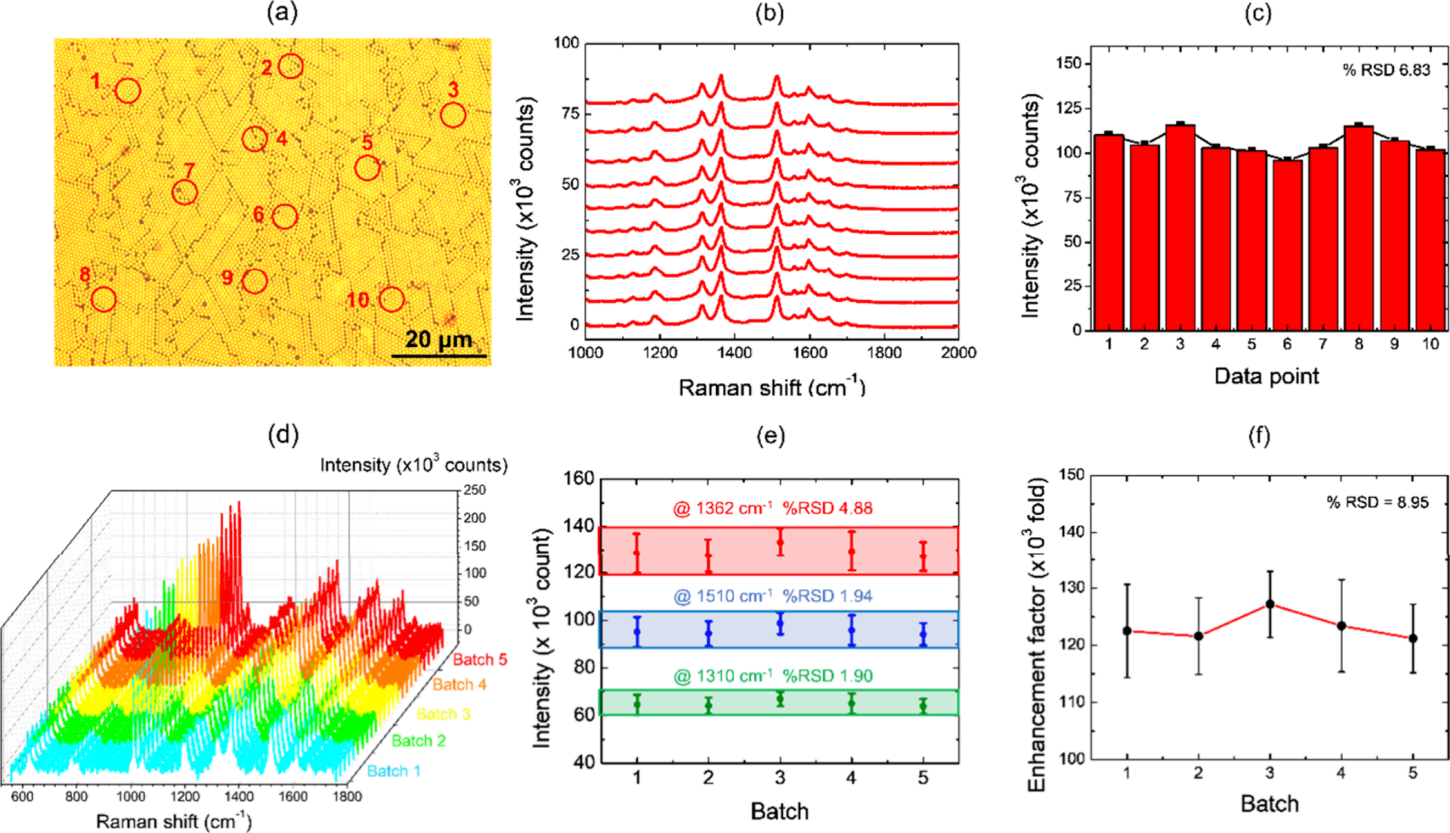
(a) Selected area for point-by-point measurement on the Au-MPS substrate prepared with PS 700 nm, (b) measured Raman spectra of 10^–5^ M R6G, and (c) point-by-point Raman intensity variation of the 1510 cm^–1^ peak with a %RSD of 6.83. (d) SERS spectra of 10^–5^ M R6G from 5 batches of samples; (e) corresponding variation of Raman intensities at 1310, 1362, and 1510 cm^–1^ from 5 batches of samples; and (f) enhancement factor of 10^–5^ M R6G from 5 batches of samples with a %RSD of 8.95.

Figure 8d,e illustrates the batch-to-batch reproducibility of the Au-MPS substrates based on their SERS response. SERS spectra collected from five independently fabricated substrates show consistent spectral profiles and stable Raman peak intensities. The relative standard deviations are approximately 1.90–4.88% for the characteristic Raman peaks (Figure 8d) and about 8.95% for the enhancement factor (Figure 8e), indicating a reliable and reproducible SERS performance.

To verify the applicability of the Au-MPS substrate for real-world detection, paraquat was selected as a target analyte. A paraquat solution was diluted to achieve various concentrations from 10^–2^ to 10^–6^ M. A 2 μL drop of each dilution was dropped on the Au-MPS substrate and dried under ambient conditions. Figure 9a shows the SERS spectra of paraquat with different concentrations. All characteristics peaks of paraquat are clearly observed, including C–N stretching (841 cm^–1^), C=C bending (1192 cm^–1^), C–C structural distortion (1300 cm^–1^), and C=N stretching (1653 cm^–1^).^31^ The SERS intensity at 1653 cm^–1^ was plotted as a function of concentration in Figure 9b. The linear plot shows a good correlation with the *R* ^2^ value of 0.98, and the LOD was calculated to be 2.54 × 10^–6^ M with the highest EF value of 3.6 × 10^3^ fold.

**Figure 9:**
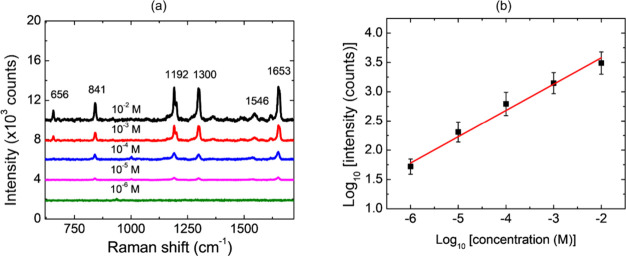
(a) SERS spectra of paraquat and (b) averaged Raman intensities at 1653 cm^–1^ plotted as a logarithmic function of paraquat concentration.

Compared with previously reported SERS substrates for paraquat detection, as shown in Table 3, the developed Au-MPS substrate exhibits a distinctive balance between plasmonic performance, surface wettability, and fabrication scalability. The integration of gold-coated monolayer spheres with a highly hydrophilic interface enables homogeneous droplet spreading and uniform molecular adsorption, leading to consistent SERS signal generation across centimeter-scale sensing regions. The periodic hexagonal close-packed configuration promotes well-defined plasmonic coupling and localized field enhancement, which collectively contribute to a highly uniform signal response and reliable measurement reproducibility.

**Table 3:** Performance Comparison of Reported SERS Substrates for Paraquat Detection

SERS substrate	LOD	EF (fold)	ref
3D laser-engraved pattern decorated with gold nanoparticles	∼10^–8^ M	∼10^9^	^31^
single-layered silver thin film	∼10^–7^ M	∼10^7^	^32^
silver cavity arrays	∼10^–7^ M		^33^
gold cactus-liked nanoparticles	∼10^–6^ M		^34^
gold-coated monolayer polystyrene spheres	∼10^–6^ M	∼10^3^	this work

Despite the detection limit achieved in this work being within the micromolar range, which may not surpass the most optimized multilayer or hybrid SERS structures, the strength of the Au-MPS design lies in its simplicity, reproducibility, and functional versatility. Its superhydrophilic nature ensures uniform analyte distribution without additional surface modification, while the doctor-blade-assisted colloidal lithography process allows for scalable, transparent, and cost-effective fabrication. These advantages make the substrates particularly suitable for practical SERS applications.

Overall, the Au-MPS substrate represents a balanced SERS platform that combines reliable optical enhancement with controllable surface wetting, offering a realistic pathway toward reproducible and field-deployable chemical and biosensing devices.

## 4 Conclusions and Outlook

In this study, we present a superhydrophilic Au-MPS SERS substrate. The substrate is fabricated using a doctor-blade-assisted colloidal lithography method, followed by gold sputter deposition. This approach enables the formation of a large-area uniform monolayer with a tunable nanostructural morphology.

Comprehensive characterization reveals that the Au-MPS substrate exhibits unique optical absorbance behavior, resulting from localized plasmonic resonances and geometric configuration. SERS measurements using R6G and the herbicide paraquat as a probe molecule demonstrate strong Raman signal enhancement, with a maximum enhancement factor of at least 3 orders of magnitude and an LOD in the micromolar range. Although the detection limit is moderate compared with those of the most optimized multilayer or hybrid SERS structures, the present design provides significant advantages in uniform signal distribution, reproducibility, and scalable fabrication.

Notably, the Au-MPS substrate offers a scalable and transparent alternative to conventional silicon-based SERS platforms, which are typically limited to reflection-mode operation. The structural and optical versatility of the Au-MPS design enables its use in both transmission and reflection modes, paving the way for broader sensor integration. The superhydrophilic surface further ensures homogeneous analyte adsorption, minimizing the coffee-ring effect and enhancing the measurement reliability across large sensing areas.

Future development of this platform could involve the implementation of multilayer sphere assemblies or integration with flexible or stretchable substrates to support wearable or portable sensing applications. Further emphasis should also be placed on enhancing the environmental stability, reproducibility, and batch-to-batch consistency of the substrates. Overall, this work demonstrates a balanced SERS platform that emphasizes practical reliability and scalable manufacturing while maintaining sufficient sensitivity for real-world chemical and biosensing applications.

## Supplementary Material



## Data Availability

The data supporting the findings of this study are available within the article and its Supporting Information.

## Notes

Simulated geometry used for the finite-element method (FEM) simulation of Au-MPS (Figure S1); comparison of SERS spectra acquired under different excitation wavelengths (Figure S2); optical images of five independently fabricated films (Figure S3); normalized absorbance spectra of Au-MPS structures with different gold thicknesses (Figure S4); water contact angle (WCA) measurements of a sputtered Au film on a silicon wafer and an Au-MPS sample (Figure S5); non-normalized and normalized optical absorbance spectra of Au-MPS substrates (Figure S6); stability test of the fabricated film substrates (Figure S7) (PDF)

## †

K.A. is deceased.
