# Supplementary material for: A Superhydrophilic Au-Coated Monolayer Polystyrene Sphere Substrate for Uniform Surface-Enhanced Raman Spectroscopy
Source: ACS Omega. 2026 Apr 16;11(16):23755–64. doi: 10.1021/acsomega.5c08273 (PMC13130117; doi:10.1021/acsomega.5c08273)
Supplement: Supplementary file 1 [file ao5c08273_si_001.pdf]

Supporting Information

Superhydrophilic Au-Coated Monolayer Polystyrene Sphere Substrate for Uniform Surface-Enhanced Raman Spectroscopy

Kittidhaj Dhanasiwawong, Kruawan Wongpanya*, Tossaporn Lertvanithphol,

Sakdinan Jantarachote, Kanin Aungskunsiri†, Mati Horprathum*

National Electronics and Computer Technology Center, 112 Thailand Science Park, Phahonyothin Road, Khlong Nueng, Khlong Luang, Pathum Thani, 12120 Thailand

**Corresponding Authors: kruawan.wongpanya@nectec.or.th; mati.horprathum@nectec.or.th*

† *Deceased*

Simulated geometry used for finite element method (FEM) simulation of Au-MPS

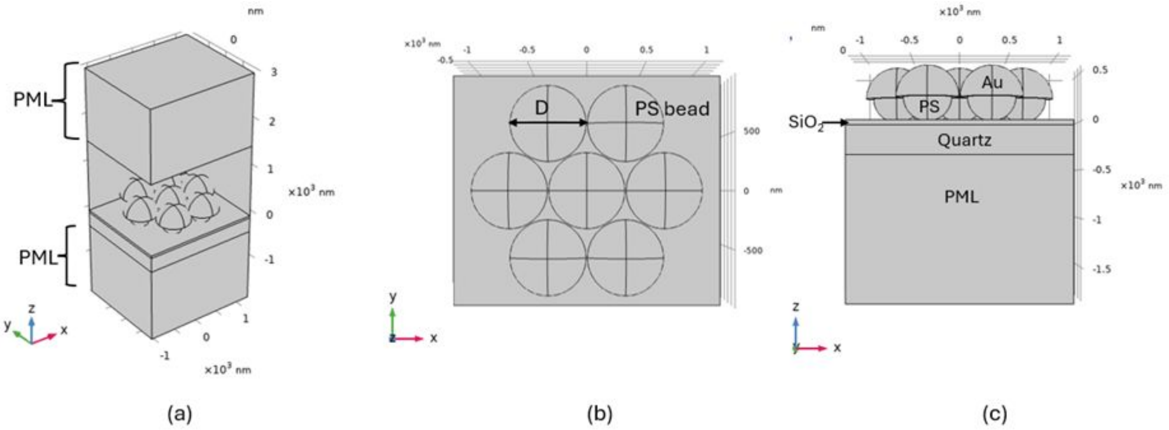

Figure S1. (a) Three-dimensional unit cell model of an Au–MPS structure used in the finite element method (FEM) simulation, showing the perfectly matched layers (PMLs) and material stack configuration. (b) Top view of the periodic unit cell in the xy-plane. (c) Cross-sectional view in the zx-plane illustrating the layered structure and port configuration.

Laser wavelength selection for Raman measurement

To select an appropriate excitation wavelength for Raman measurements, SERS spectra were recorded using excitation wavelengths of 532, 633, and 785 nm, as shown in Figure S2. Although higher overall Raman intensities were observed for the 532 and 633 nm excitations, these spectra exhibited a significantly elevated background and increased noise levels, which reduced spectral clarity and hindered reliable peak identification.

In contrast, the spectrum obtained using the 785 nm excitation wavelength showed the highest signal-to-noise ratio among the tested wavelengths. This provided clearer Raman features and more reliable spectral interpretation.

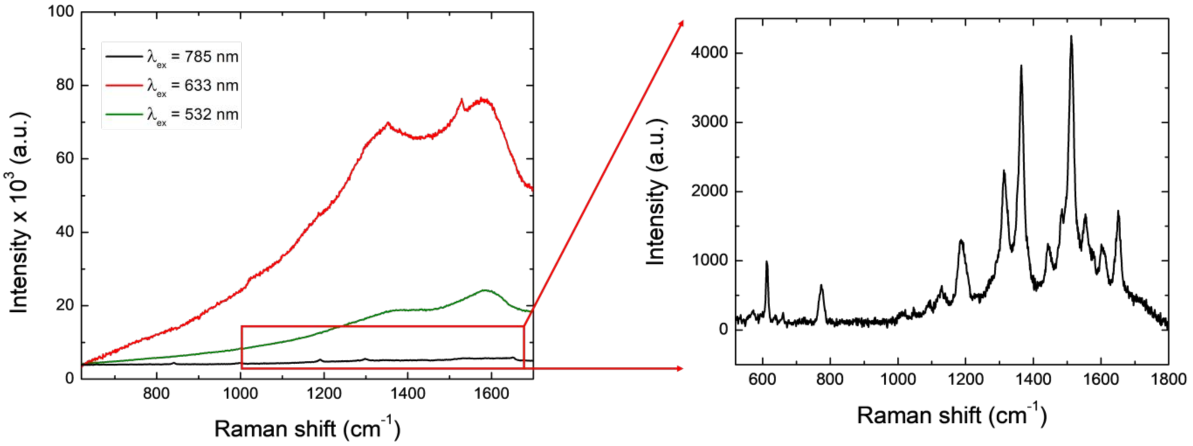

Figure S2. Comparison of SERS spectra acquired under different excitation wavelengths (532, 633, and 785 nm), illustrating the trade-off between Raman intensity and background noise.

Reproducibility of the fabricated film substrates

Five independent films were fabricated under identical conditions to evaluate the reproducibility of the preparation process. Optical images of each film are shown in Figure S3. A fixed 7 × 7 mm^2 square region of interest (ROI) at the center of each film was analyzed using ImageJ. The mean gray-intensity values within these ROIs were used to quantify the optical uniformity of the films. The reproducibility was calculated according to:

$$\text{Reproducibility (\%)} = \left(1 - \frac{\text{SD}}{\text{Mean}}\right) \times 100$$

The calculated reproducibility of five independent films was found to be 88.92 %, indicating good consistency and uniform coating quality among the independently prepared films.

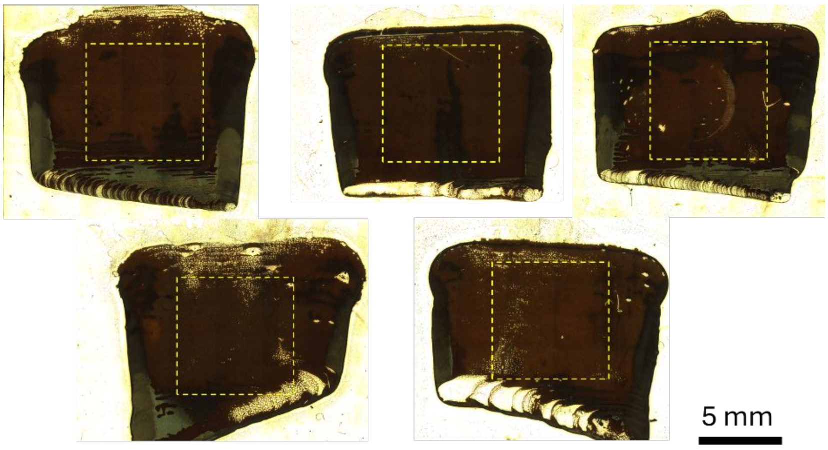

Figure S3. Optical images of five independently fabricated film substrates, showing the region of interest (ROI) used for reproducibility analysis.

Au thickness selection

Figure S4 shows the simulation normalized absorbance spectra of the Au-MPS structures with different gold thicknesses ranging from 50 to 90 nm. As the gold thickness increases, a gradual evolution of the plasmonic resonance features is observed, including a redshift and improved definition of the localized surface plasmon resonance (LSPR) band in the near-infrared region.

Among the investigated thicknesses, the Au layer with a thickness of 75 nm exhibits the most distinct and well-defined LSPR features, particularly in the spectral region relevant to the Raman excitation wavelength used in this study. Thinner films (<70 nm) show less pronounced resonance features, while thicker films (>80 nm) result in broadened absorption profiles, likely due to increased damping effects.

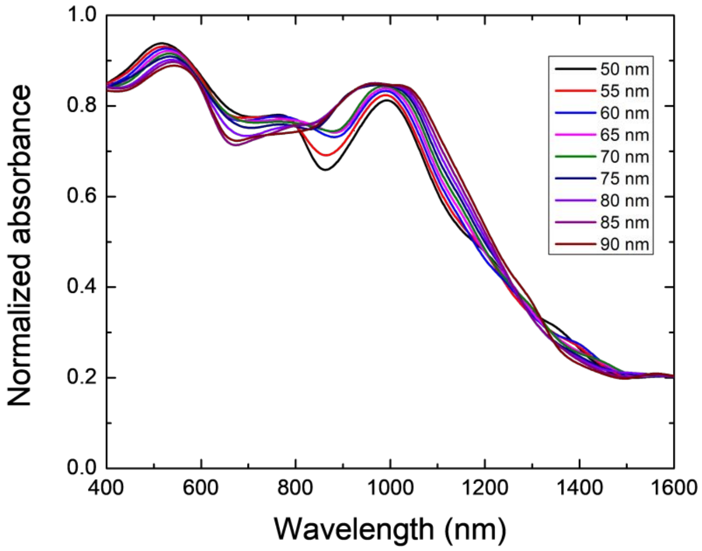

Figure S4. Simulated normalized optical absorbance spectra of Au–MPS structures with different gold thicknesses ranging from 50 to 90 nm, showing the evolution of plasmonic resonance features.

Water contact angle measurement

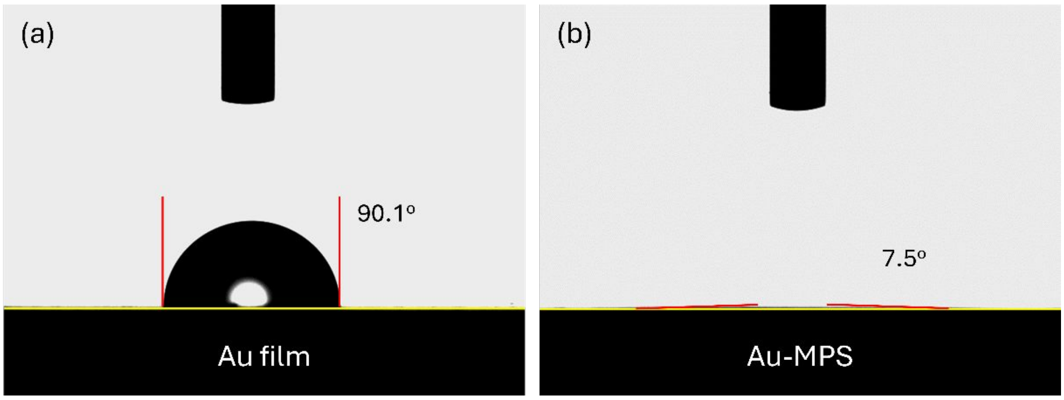

Figure S5. Water contact angle (WCA) measurements of (a) a sputtered Au film on a silicon wafer and (b) an Au–MPS sample, demonstrating the difference in surface wettability.

Non-normalized (left) and normalized (right) optical absorbance spectra of Au–MPS substrates

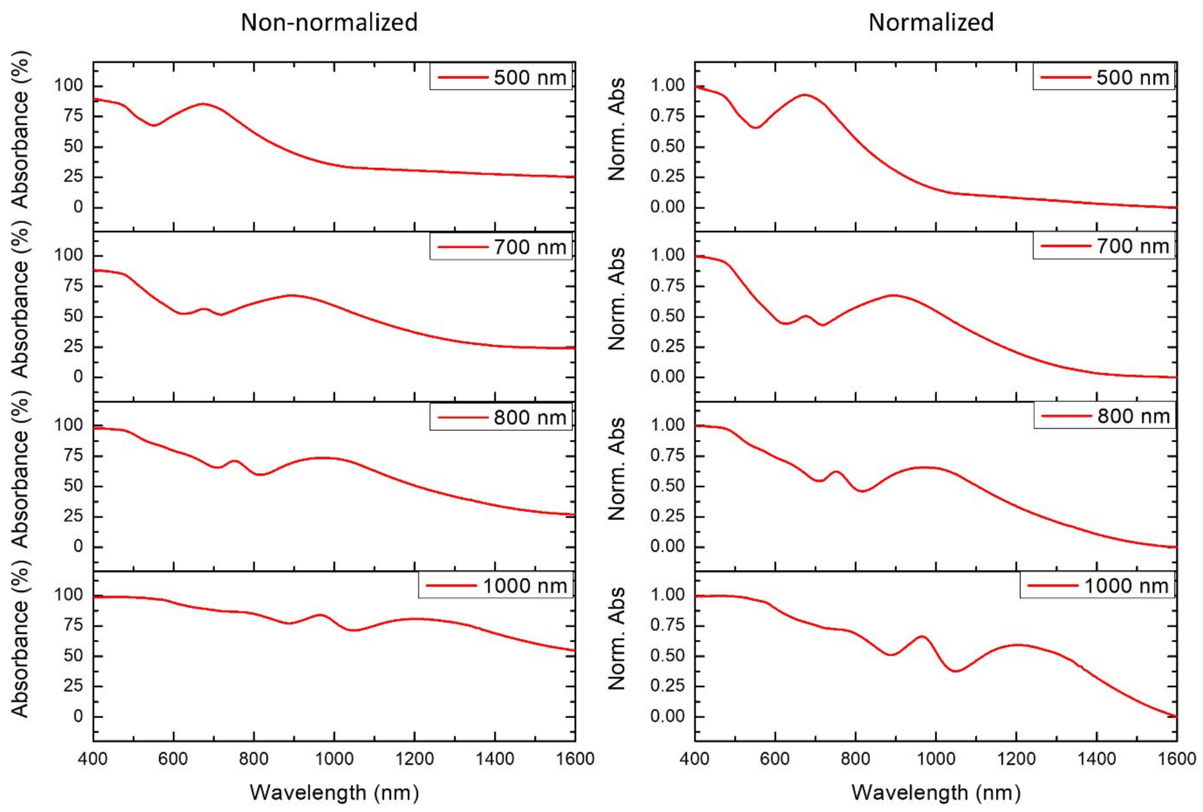

Figure S6. Non-normalized (left) and normalized (right) optical absorbance spectra of Au–MPS substrates. The non-normalized spectra exhibit the same characteristic absorption features and trends as the normalized spectra, confirming that the observed plasmonic resonances are intrinsic to the Au–MPS structures and not artifacts of the normalization process.

Stability test of the fabricated film substrates

The long-term stability of the SERS substrate was evaluated by measuring its SERS performance after extended storage, as this parameter directly reflects its practical usability.

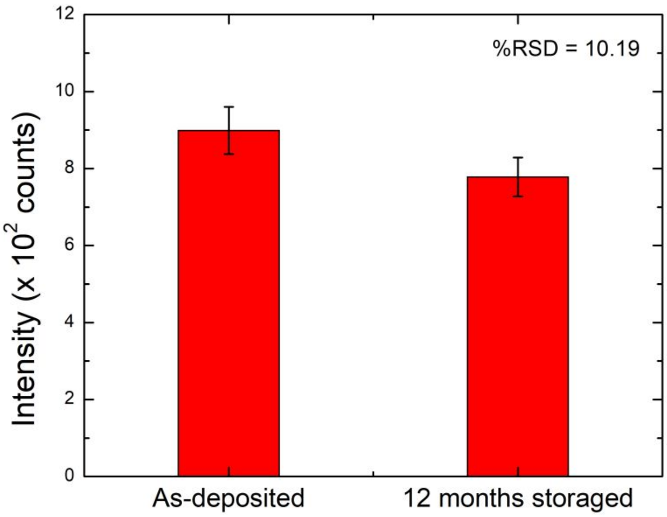

Figure S7. SERS intensity of 10^{-5} M R6G measured on the as-deposited film and the same film after 12 months of storage, demonstrating the long-term stability of the substrate.
